# The pretemporal approach to anterolateral midbrain cavernous malformations

**DOI:** 10.3171/2019.10.FocusVid.19435

**Published:** 2019-10-01

**Authors:** Daniel D. Cavalcanti, Vance Fredrickson, Paulo Niemeyer Filho

**Affiliations:** 1 Department of Departments of Neurosurgery and Radiology, New York University School of Medicine, New York, New York;; 2 Department of Neurological Surgery, Keck School of Medicine, University of Southern California, Los Angeles, California; and; 3 Department of Neurosurgery, Paulo Niemeyer State Brain Institute, Rio de Janeiro, Rio de Janeiro, Brazil

**Keywords:** brainstem, cavernous malformation, microsurgery, pretemporal approach, video

## Abstract

Operating on the anterolateral midbrain is challenging due to limited surgical freedom provided by classic approaches and restraints imposed by the basilar artery apex and branches, their perforators, and the oculomotor nerve (Abla et al., 2011; Bricolo and Turazzi, 1995; Cavalcanti et al., 2016).

This video demonstrates the benefits provided by the pretemporal approach for resection of an anterolateral mesencephalic cavernous malformation (Chaddad-Neto et al., 2014; de Oliveira et al., 1995). Four steps are well demonstrated in the video: 1) section of temporal pole veins to the sphenoparietal sinus; 2) division of arachnoid attaching the oculomotor nerve to the tentorial edge and uncus; 3) releasing the arachnoid between the anterior choroidal artery and uncus; and 4) following the oculomotor nerve to its origin.

The video can be found here: https://youtu.be/7ZuK-ewNo6w.

## Transcript

This is the history of a 52-year-old female with two acute episodes of right-side weakness, one in 2012 and one in 2014, and numbness in right arm and leg, as well as in the left hemiface and unsteadiness. The physical exam was significant for a motor strength of 4– in both right upper and right lower extremities, tremor and dysmetria on the left, and ataxia.

Preoperative MRI **(0:53)** show a large cavernous malformation in the left anterolateral midbrain, occupying both cerebral peduncle and tegmentum. The lesion abuts the pial plane of both the ambiens and crural cisterns. It is a Zabramski type II lesion, with the classic popcorn sign and the low-signal ring of blooming in T2 sequences.

Landmarks in a cadaver model **(1:19)**. A frontotemporal incision is carried out beginning in the superior edge of the zygomatic arch, curving anteriorly, close to the midpupillary line, ending just behind the hairline. An interfascial dissection is completed **(1:33)** in order to avoid damage to the frontal branch of the facial nerve. A basal fronto-temporo-sphenoidal craniotomy is completed **(1:42)**, with the keyhole just behind the frontozygomatic suture.

The hatched area in green **(1:48)** demonstrates the area of exposure of this approach on the anterolateral midbrain and pontomesencephalic junction. Tracking down the oculomotor nerve to its origin takes us to the pontomesencephalic junction, just between the proximal segments of the SCA (superior cerebellar artery) and PCA (posterior cerebral artery). The pretemporal approach provides robust exposure of the crural and anterior ambiens cisterns.

The patient is placed supine on the operative table **(2:14)**, the head of the bed is raised to 30°, and a small cushion is placed under the ipsilateral shoulder. The head is then extended, rotated 30° to the contralateral side, and minimal lateral flexion is applied. A frontotemporal incision is carried out **(2:33)**, starting at the superior edge of the zygomatic arch, curving posteriorly and then anteriorly towards the midpupillary line. An interfascial dissection is carried out, the temporal muscle is incised, retracted inferiorly, and a temporal muscle cuff is left along the superior temporal line. A fronto-temporo-sphenoidal craniotomy **(2:55)** is carried out as previously mentioned. The dura is opened in a C-shaped fashion.

The dissection is started **(3:03)** by opening the Sylvian fissure. Then the carotid and chiasmatic cisterns are opened. And then the dissection continues laterally to the internal carotid artery, opening the carotid-oculomotor triangle. All the arachnoid **(3:32)** between the temporal lobe and the tentorial edge is divided. Bridging veins between the temporal pole **(3:40)** and the sphenoparietal sinus are coagulated and divided. Extending the opening of the chiasmatic cistern provides a better exposure of the opticocarotid triangle and the Liliequist membrane. The cistern of the lamina terminalis is also opened. The arachnoid binding the third nerve to the uncus **(4:30)** is wide opened, allowing better mobility of the temporal lobe. Tracking down the track of the third nerve takes us to the ventral pontomesencephalic junction, just between the course of the proximal segments of the posterior cerebral artery and the superior cerebellar artery. It is really important to wide open the crural cistern **(5:24)**, by releasing the arachnoid membrane between the anterior choroidal artery and the uncus.

The optimal positioning of head and trunk, and the wide opening of all the exposed cisterns, dispense the need for fixed retraction.

The left A1 segment and the optic chiasm are seen **(5:58)**. The left posterior communicating artery and the anterior choroidal artery are seen in the crural cistern **(6:09)**. Image guidance is crucial to locate the cavernous malformation **(6:24)**. The apparent origin of the third cranial nerve is seen **(6:34)** and the anterolateral midbrain is exposed. The yellowish hemosiderin stain is seen on the pial surface of the midbrain. The combination of the transsylvian, subfrontal, and temporopolar dissections **(7:11)** gives an optimal exposure of this cavernous malformation. It is possible to see now **(7:18)** this cavernous malformation abutting the pial surface of the anterolateral midbrain.

The neurotomy **(7:24)** is performed just between the SCA and PCA. We initially enlarge the neurotomy by moving nonviable tissue far from the corticospinal tract. For a large lesion like this one, a piecemeal resection is preferred. A large fragment is seen removed **(8:10)**. Image guidance and monitoring and constantly rechecked.

The dissection plane is moved medially **(8:43)**, and old hematoma is evacuated. Fragments in different stages of evolution are resected. A large pale fragment is seen removed **(9:13)**. The 1-mm cup forceps is one of the main tools used to resect this cavernous malformation. A flowable hemostatic matrix **(9:28)** is used to perfectly fill the deep and complex operative bed. After aspirating most of the hemostatic agent, we continue the dissection in different directions. More fragments are seen removed **(9:52)**, and bipolar coagulation in minimal power is carried out.

Motor exam worsened initially, down to 3 out of 5, and she had diplopia **(10:06)**. Both improved after 3 months of follow-up. Three-month follow-up MRI of the brain **(10:12)** showed complete resection of the midbrain cavernous malformation.
